# Sleep spindle alterations in patients with Parkinson's disease

**DOI:** 10.3389/fnhum.2015.00233

**Published:** 2015-05-01

**Authors:** Julie A. E. Christensen, Miki Nikolic, Simon C. Warby, Henriette Koch, Marielle Zoetmulder, Rune Frandsen, Keivan K. Moghadam, Helge B. D. Sorensen, Emmanuel Mignot, Poul J. Jennum

**Affiliations:** ^1^Biomedical Engineering, Department of Electrical Engineering, Technical University of DenmarkKongens Lyngby, Denmark; ^2^Danish Center for Sleep Medicine, Department of Clinical Neurophysiology, Glostrup University HospitalGlostrup, Denmark; ^3^Stanford Center for Sleep Sciences and Medicine, Psychiatry and Behavioral Sciences, Stanford UniversityPalo Alto, CA, USA; ^4^Center for Advanced Research in Sleep Medicine, Sacré-Coeur Hospital of Montréal, University of MontréalMontréal, QC, Canada; ^5^Department of Neurology, Bispebjerg HospitalCopenhagen, Denmark; ^6^Department of Biomedical and Neuromotor Sciences (DIBINEM), University of BolognaBologna, Italy; ^7^Center for Healthy Ageing, University of CopenhagenCopenhagen, Denmark

**Keywords:** Parkinson's disease, sleep spindle morphology, EEG, neurodegeneration, biomarker

## Abstract

The aim of this study was to identify changes of sleep spindles (SS) in the EEG of patients with Parkinson's disease (PD). Five sleep experts manually identified SS at a central scalp location (C3-A2) in 15 PD and 15 age- and sex-matched control subjects. Each SS was given a confidence score, and by using a group consensus rule, 901 SS were identified and characterized by their (1) duration, (2) oscillation frequency, (3) maximum peak-to-peak amplitude, (4) percent-to-peak amplitude, and (5) density. Between-group comparisons were made for all SS characteristics computed, and significant changes for PD patients vs. control subjects were found for duration, oscillation frequency, maximum peak-to-peak amplitude and density. Specifically, SS density was lower, duration was longer, oscillation frequency slower and maximum peak-to-peak amplitude higher in patients vs. controls. We also computed inter-expert reliability in SS scoring and found a significantly lower reliability in scoring definite SS in patients when compared to controls. How neurodegeneration in PD could influence SS characteristics is discussed. We also note that the SS morphological changes observed here may affect automatic detection of SS in patients with PD or other neurodegenerative disorders (NDDs).

## Introduction

Parkinson's disease (PD) is a neurodegenerative disorder (NDD) characterized primarily by motor symptoms, including bradykinesia, rigidity, postural instability, and tremor. Although the disease process in PD is not restricted to a specific brain area, these symptoms are mostly caused by the loss of dopaminergic neurons in the substantia nigra pars compacta resulting in a reduction or depletion of dopamine (Galvin et al., 2001). Lewy body aggregations of alpha-synuclein in the brain are a central feature of PD pathology (Galvin et al., 2001). These inclusions typically start in caudal areas of the brain and progress anteriorly (Braak et al., 2003), and may take place years prior to involvement of the substantia nigra and associated development of motor symptoms. Specifically, Braak et al.'s PD staging is based on Lewy-body distribution, which rise from the dorsal motor nucleus of the vague nerve in the medulla and in the olfactory bulb (stage 1) emerging through the subceruleus-ceruleus complex and the magnocellularis reticular nucleus (stage 2), the substantia nigra, the pedenculopontine nucleus and the amygdala (stage 3), the temporal mesocortex (stage 4), and finally reaching the neocortex (stage 5 and 6). Stage 1 and 2 were considered as pre-Parkinsonian states, stage 3 and 4 as Parkinsonian states and 5 and 6 as late-Parkinsonian states (Braak et al., 2003).

In addition to the motor manifestations that define PD, non-motor symptoms such as sleep problems, depression, dementia and attention deficit (Chaudhuri et al., 2011, 2006), autonomic symptoms as abnormal heart rate variability (Sorensen et al., 2012, 2013) and gastrointestinal symptoms such as nausea and constipation (Garcia-Ruiz et al., 2014) are all well known in patients with PD. Stating the presence of at least two of the four motor symptoms resting tremor, bradykinesia, rigidity, and postural imbalance typically makes the clinical diagnosis of PD, although it has been indicated that the pathological changes in the striatal dopaminergic system develop several years before the clinical appearance of PD. Further development of the pathology may result in Lewy Body Dementia.

Twenty years ago, it was discovered that idiopathic rapid eye movement (REM) sleep behavior disorder (iRBD) is closely related to Parkinsonism (Schenck et al., 1996, 2013a; Salawu et al., 2010). Indeed, the presence of iRBD, even without the presence of motor or cognitive complaints, confers a significant risk of conversion into synnucleinopathies including PD (Iranzo, 2011; Schenck et al., 2013b). The diagnosis of RBD requires complaints or an anamnesis describing dream enactment behaviors as well as a manifestation of REM sleep without atonia (RSWA) as measured by polysomnography (PSG) (Stevens and Comella, 2013; American Academy of Sleep Medicine, 2014). The idiopathic form of RBD (iRBD) is diagnosed when no concurrent neurological disease is found, and International classification of Sleep Disorders criteria for RBD are met (Stevens and Comella, 2013; American Academy of Sleep Medicine, 2014). Specifically, measures of RSWA (Postuma et al., 2010; Kempfner et al., 2013), slow wave characteristics (Latreille et al., 2011), sleep stability and differences in electroencephalographic (EEG) or electrooculographic micro- and macro-sleep patterns have been investigated in patients with iRBD and/or PD (Christensen et al., 2012, 2013, 2014b).

Reduced sleep spindle (SS) density and activity have been identified in patients with PD and iRBD (Puca et al., 1973; Myslobodsky et al., 1982; Emser et al., 1988; Comella et al., 1993; Christensen et al., 2014a; Latreille et al., 2015). SS are generated by a complex interaction involving thalamic, limbic, and cortical areas. A di-synaptic circuit between thalamic reticular neurons and thalamocortical relay cells, both located in the thalamus, can spontaneously generate spindle-like oscillations, which are conveyed to the cortex by the axons of the thalamocortical relay cells. These cells receive feedback from cortical pyramidal cells as well as input from pre-thalamic fibers originating from the brainstem and posterior hypothalamus (Steriade et al., 1993; Steriade and Timofeev, 2003). As such the thalamus holds a primary role in generating and controlling SS. SS have been reported to have a gating role with regard to the flow of thalamic sensory input, and thus may have a sleep-preserving role (De Gennaro and Ferrara, 2003). Also, several studies have reported SS to have an important role in memory consolidation, synaptic plasticity and cognition (Steriade and Timofeev, 2003; Schabus et al., 2006; Fogel and Smith, 2011; Fogel et al., 2012; Latreille et al., 2015). The formation of SS begins in the infant brain (De Gennaro and Ferrara, 2003), but SS characteristics such as density and amplitude change with age (Nicolas et al., 2001; De Gennaro and Ferrara, 2003), suggesting that SS play an important role in normal cognitive functioning.

Although a reduction in SS density is not specific to PD, SS and other EEG features may be potential useful as biomarkers of disease progression or therapeutic efficacy in PD and other NDDs (Nguyen et al., 2010; Leiser et al., 2011; Micanovic and Pal, 2014). However, the identification of SS is a difficult task; studies assessing inter-scorer variance in normal sleep have shown significant variance in SS identification, both between human experts and between automated SS detectors (Warby et al., 2014; Wendt et al., 2014). SS identification and characterization in pathological sleep is not well studied, but previous evidence suggests that SS may have different characteristics in PD patients (Latreille et al., 2015), and therefore may interfere with traditional sleep staging in patients (Comella et al., 1993; Jensen et al., 2010; Christensen et al., 2014b; Koch et al., 2014).

In this study, we aimed to identify changes in SS density and specific morphological characteristics of SS in patients with PD. Since five sleep experts identified SS independently, we were also able to assess inter-expert variation of SS identification in EEG of patients and controls. By identifying specific changes in SS characteristics, we aimed to better understand the mechanism and to what extent the neurodegenerative progress influences SS characteristics, also identifying specific spindle features that may be useful as prognostic biomarkers of disease. A secondary aim was to help guide the specialized development of automatic SS detectors to be used on EEG from patients with NDDs.

## Materials and methods

### Subjects and recordings

Polysomnographic (PSG) EEG data from 15 patients with PD and 15 sex- and age-matched control subjects with no history of movement disorder, dream-enacting behavior or other previously diagnosed sleep disorders were included in this study. The subjects were all recruited from the Danish Center for Sleep Medicine (DCSM) in the Department of Clinical Neurophysiology, Glostrup University Hospital in Denmark. All patients were evaluated by a movement specialist with a comprehensive medical and medication history and a PSG analyzed according to the American Academy of Sleep Medicine (AASM) standard (Iber et al., 2007). The diagnostic certainty for PD at Danish neurological departments has been reported to be 82% (Wermuth et al., 2012). None of the PD patients had dementia at inclusion, but one of the patients with PD later developed Multiple System Atrophy (MSA), indicated as the Parkinsonian type (MSA-P) as the patient had predominating PD-like symptoms. Subjects were excluded from the study if they were taking medications known to effect sleep (antidepressants, antipsychotics, hypnotics). However, dopaminergic treatments were permitted despite their potential effect on vigilance and SS characteristics (Puca et al., 1973; Micallef et al., 2009). In addition to ethical concerns regarding discontinuing dopaminergic treatment in these subjects, we wanted to avoid deleterious discontinuation effects on the PSG, as well as unpleasant and negative motor effects that could interfere with the study. The quality of each PSG recording was individually examined, and recordings with disconnections or significant amounts of signal artifact were not included. Demographic data and PSG variables for the two groups are seen in Table 1.

**Table 1:** **Demographic and PSG data for the two groups studied**.

**Characteristics**	**PD patients**	**Controls**	***P***
Total counts (Male/Female)	15 (7/8)	15 (7/8)	–
Age (Years)	62.7 ± 5.8	62.9 ± 5.9	0.90
BMI (kg/m^2^)	25.3 ± 3.5	22.1 ± 2.5	0.02
Disease duration (years)	6.7 ± 4.5	NA	–
Hoehn and Yahr stage	2.0 ± 1.2	NA	–
UPDRS part III “on”	20.9 ± 7.0	NA	–
ACE	90.2 ± 4.8	NA	–
Levodopa equivalent dosage (mg)	621.1 ± 301.5	NA	–
Levodopa use [n (%)]	10 (67)	NA	–
Dopamine agonist use [n (%)]	14 (93)	NA	–
Sleep efficiency (%)	79.7 ± 14.1	87.1 ± 8.4	0.09
Time in bed (min)	448.1 ± 82.0	499.6 ± 63.7	0.07
LM index (number/hour)	31.8 ± 34.8	30.4 ± 35.3	0.91

*BMI, Body Mass Index; UPDRS, Unified Parkinson's disease rating scale; ACE, Addenbrooke's cognitive examination; LM, Leg movements*.

### Manual labeling of sleep spindles

For each subject, eight blocks of five consecutive epochs of non-REM sleep stage 2 (N2) of 30-s duration were selected randomly from the PSG recording in between lights off and lights on. The blocks were randomly chosen and ranked by use of Matlab's *randsample*-function. One-by-one and in the prioritized order, the blocks were visually checked for major movements or other contaminating artifacts. The first eight artifact-free blocks were chosen as the ones to be scored for SS. A total of five independent sleep experts identified SS in these blocks, where only the C3-A2 EEG derivation was visible. The signals were filtered with a notch filter at 50 Hz and a band-pass filter with cutoff frequencies at 0.3 Hz and 35 Hz, as indicated by AASM standards (Iber et al., 2007). All analyzed signals had a sampling frequency of 256 Hz. The experts assigned a confidence score to each identified spindle, to indicate the amount of confidence in the identification (as described previously in Warby et al., 2014). In this way, each SS was given a confidence weighting of 1 for “definitely SS,” 0.75 for “probably a SS” and 0.5 for “maybe a SS.”

The scoring procedure was performed in a Matlab-based software program “EEG viewer” developed by MN at DCSM. The program mimics a standard sleep scoring program in a clinical setting, and includes the standard features so the experts have the same opportunities to view and navigate the PSG data as they are used to when analyzing sleep in the clinic. The program ensures that if an epoch to be scored does not have any marked SS, the expert is required to click a box saying “no spindles in current epoch.” This ensures that the total of 40 epochs of N2 sleep per subject was analyzed by each expert. The experts were blinded for which group the subjects belong to.

The final SS identifications used for morphology measures were defined using the group consensus rule described in Warby et al. (2014). Spindle identifications from five different experts with weighted confidence scores for each SS were averaged at each sample point and aggregated into a single consensus. Sample points that had an average score of higher than the group consensus threshold *T_gc_* = 0.25 were included in the final group consensus, and the morphology measures were computed on these group consensus SS. It was decided to use *T_gc_* = 0.25 as this was found to be the best in Warby et al. (2014).

### Spindle characteristics and between group comparisons

The morphology of the identified SS was characterized by their (1) duration, (2) oscillation frequency, (3) maximum peak-to-peak amplitude, (4) percent-to-peak amplitude, and (5) SS density per minute; all of which are well-evaluated elsewhere (Warby et al., 2014). The morphology measures were all computed using Matlab 2013b. Before any of the measures were computed, the central EEG signal was filtered forward and reverse with (1) a notching filter with the notch at 50 Hz and a bandwidth of 50/35 Hz (at −3 dB) and (2) a 4th order Butterworth band-pass filter with cut off frequencies (−3 dB) at 0.3 Hz and 35 Hz.

For each SS the duration was computed in seconds as

$$dur=\frac{\#\;samples}{f_{s}},$$

where *f_s_* = 256 Hz is the sampling frequency and *# samples* defines the number of samples. The samples were consecutive and obeyed the consensus rule. The oscillation frequency was defined in Hz and was for each SS estimated as

$$f_{osc}=\frac{K}{2\;\cdot \;dur},$$

where *K* defines the total number of extrema points detected using Matlab's *findpeaks*-function applied on a 5-point moving average smoothed version of the SS signal and with a minimum peak-to-peak distance of 11 samples. The maximum points were found by applying the *findpeaks*-function directly, and the minima points were found by applying the function on the flipped signal, and the total number of extrema points was set as the sum of the two. These settings were chosen, as they were considered best for estimating the *f_osc_* when visually investigating numerous randomly selected examples of SS. The maximum peak-to-peak amplitude was for each SS estimated as

$$A_{p2p}=\max\left(\left|A_{e}\left(k\;+\;1\right)-A_{e}(k)\right|\right),\;k=1,\;2,\ldots ,\;K-1,$$

where *A_e_* is a vector holding the amplitude values for each of the *K* detected extrema points. To investigate the influence on SS from K-complexes or delta waves, the maximum peak-to-peak amplitude was estimated twice for each SS; once without any further frequency filtering of the data, and once where the data was forward and reverse filtered with a 10th order high-pass filter with cut off frequency (−3 dB) at 4 Hz to remove low frequency, high amplitude waves that may interfere with the peak-to-peak calculation. The percent-to-peak amplitude gives a simple measure between 0 and 1 of the symmetry of the spindle and it was computed for each SS as

$$Sym=\frac{\#\;samples\;before\;point\;of\;A_{p2p}}{\#\;samples},$$

where the point of *A_p2p_* is defined as the point between the maxima and minima delineating *A_p2p_*. Finally, the density was computed for each subject as the number of SS per minute of investigated data, described as

$$Density=\;\frac{2\;\cdot \;\#\;SS}{\#\;epochs\;reviewed}.$$

The morphology measures were computed for the SS identifications for each expert, as well as for the spindles included in the group consensus. For the SS included in the group consensus, a minimum duration threshold *dur_th_* = 0.2 s was used, and resulted in the exclusion of only three spindles. This threshold is less that the minimum duration stated by the AASM scoring (0.5 s). However, others have shown that apparent spindles <0.5 s are clearly recognizable by sleep experts, and have similar characteristics to spindles >0.5 s (Warby et al., 2014). We used a minimum duration threshold of 0.2 s because we wanted to determine whether PD patients and controls have specific differences in these shorter spindles. When computing the measures for the SS identifications for each expert, all the SS were included, regardless of their confidence score and duration. Two-sided Wilcoxon rank sum tests with a significance level of α = 0.05 were used for each of the measures to test for significant differences between the two groups.

### Inter-expert reliability when scoring SS

Inter-expert reliability measures were computed for each of the 10 available expert-pairs. True positives (TP) define the number of samples where both experts have marked SS, true negatives (TN) define the number of samples where both experts have not marked SS, false positives (FP) define the number of samples where the reference-expert has not marked SS, and the other expert has and false negatives (FN) define the number of samples where the reference-expert has marked SS, but the other expert has not. For each comparison, the reliability measures were indicated as the *F*_1_-score and the Cohen's Kappa coefficient (κ). The *F*_1_-score is the harmonic mean of precision (P) and recall (R) and reaches its best value at 1 (perfect agreement) and the worst at 0 (no agreement). It is computed as

$$\begin{array}{l} F_{1}\text{-score}=\frac{2\;\cdot \;R\;\cdot \;P}{R+P},\text{where}\\ \;R=\frac{TP}{TP+FN}\text{ and }P=\frac{TP}{TP+FP}. \end{array}$$

The κ is often used to measure inter-annotator reliability as it takes the agreement occurring by chance into account. It reached its best value at 1 (perfect agreement) and worst at -1 (no agreement). It reaches 0 when accuracy is equal to what is expected by chance. It is computed as

$$\begin{array}{l} \;\kappa =\frac{\frac{TP+TN}{N}-\;Pr}{1-Pr},\text{where}\\ Pr=\frac{TP+FN}{N}\;\cdot \;\frac{TP+FP}{N}+\\ \;\left(1-\frac{TP+FN}{N}\right)\cdot \left(1-\frac{TP+FP}{N}\right), \end{array}$$

where *N* = *TP* + *TN* + *FP* + *FN* defines the total number of samples reviewed. The relative strength of agreement associated with κ can been described by the labels “poor” (κ < 0.00), “slight” (0.00 ≤ κ ≤ 0.20), “fair” (0.21 ≤ κ ≤ 0.40), “moderate” (0.41 ≤ κ ≤ 0.60), “substantial” (0.61 ≤ κ ≤ 0.80) and “almost perfect” (0.81 ≤ κ ≤ 1.00) (Landis and Koch, 1977). The *F*_1_-score and κ are symmetric regarding false detections and will therefore both yield the same regardless of which expert were used as the reference.

## Results

**Figure 1:**
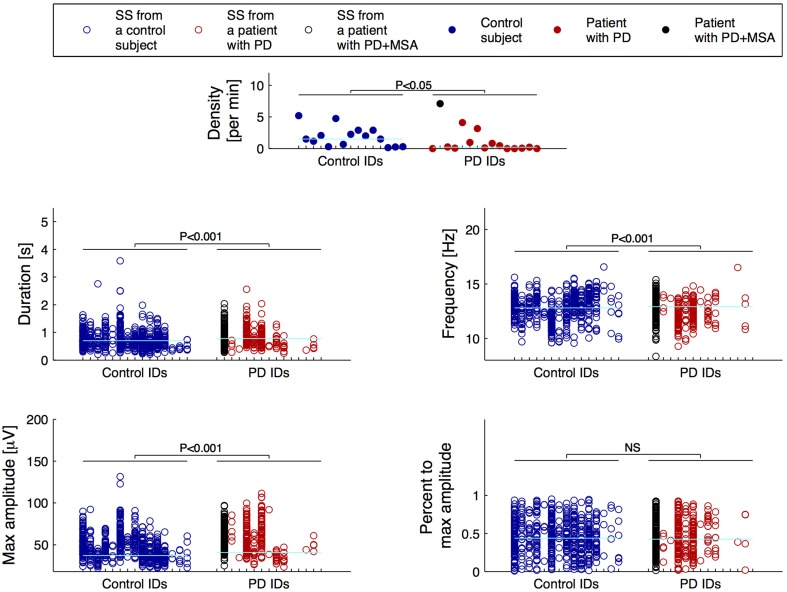
**Distributions of the morphology measures for the spindles included in the group consensus**. From left to right, the first eight IDs in both groups are females ranging from the youngest to the oldest, and the following seven IDs are males also ranging from the youngest to the oldest. One patient with Parkinson's disease (PD) later developed Multiple System Atrophy (MSA) and is indicated with black. The cyan horizontal lines indicate the group median for each of the measures.

For the SS included in the group consensus, it was found that patients with PD show SS that are significantly different from controls in terms of duration, oscillation frequency and max peak-to-peak amplitude. Additionally, patients with PD have significantly different SS density compared to controls. Specifically, it was found that patients with PD have decreased SS density (−38.17%/−0.71 SS/min), and that their SS are longer (+11.69%/+0.09 s), have a lower frequency (−2.27%/−0.29 Hz) and higher max peak-to-peak amplitude (+19.61%/9.45 μV) compared to controls (Table 2). No significant differences were identified for the symmetry measure. The maximum peak-to-peak amplitude estimated after removal of frequencies below 4 Hz was still significantly different between groups. Of note, patients with PD still showed a higher max peak-to-peak amplitude (+20.95%/9.49 μV) compared to controls. The five SS morphology measures are illustrated in Figure 1. From left to right, the eight first ID numbers in both groups are females ranging from the youngest to the oldest. The last seven IDs in both groups are males, also ranging from the youngest to the oldest. One of the patients later developed MSA and is illustrated with black.

**Table 2:** **Mean (μ) and standard deviation (σ) for the spindle characteristics found for each of the experts' identifications as well as for the spindles in the group consensus**.

**Spindle characteristic**	**Expert 1 (947 SS)**		**Expert 2 (752 SS)**		**Expert 3 (952 SS)**		**Expert 4 (282 SS)**		**Expert 5 (2135 SS)**		**Group consensus (901 SS)**		***P***
	***PD***	***C***	***PD***	***C***	***PD***	***C***	***PD***	***C***	***PD***	***C***	***PD***	***C***	
Duration [sec, μ ± σ ]	0.93 ± 0.44	0.84 ± 0.41	0.66 ± 0.29	0.67 ± 0.29	0.74 ± 0.29	0.68 ± 0.27	0.88 ± 0.20	0.77 ± 0.24	1.19 ± 0.52	1.12 ± 0.51	0.86 ± 0.35	0.77 ± 0.36	<0.002^a^^,^^c^^,^d^,^^e^^,^^GC^
Frequency [Hz, μ ± σ ]	12.38 ± 1.27	12.69 ± 1.27	12.92 ± 1.24	13.07 ± 1.11	12.45 ± 1.22	12.62 ± 1.34	12.73 ± 1.14	13.13 ± 1.00	11.69 ± 1.24	12.03 ± 1.28	12.51 ± 1.21	12.80 ± 1.23	<0.02^a^^,^^b^^,^^d^^,^^e^^,^^GC^
Max peak-to-peak amplitude [μV, μ ± σ ]	57.37 ± 17.23	48.26 ± 15.37	56.96 ± 18.14	46.88 ± 15.96	57.75 ± 17.15	48.52 ± 15.47	64.60 ± 16.68	49.95 ± 14.04	51.44 ± 18.14	45.02 ± 15.73	57.64 ± 17.34	48.19 ± 15.55	<0.001^a^^,^^b^^,^^c^^,^d^,^^e^^,^^GC^
Max peak-to-peak amplitude, after removal of frequencies < 4 Hz [μV, μ ± σ ]	53.87 ± 15.99	45.24 ± 14.17	54.38 ± 16.85	44.16 ± 14.89	54.89 ± 16.34	45.43 ± 14.18	62.40 ± 16.64	47.51 ± 13.13	46.20 ± 16.62	40.15 ± 13.92	54.78 ± 16.24	45.29 ± 14.41	<0.001^a^^,^^b^^,^^c^^,^^d^^,f,^^GC^
Percent-to-peak amplitude [μ ± σ ]	0.49 ± 0.23	0.47 ± 0.24	0.46 ± 0.23	0.46 ± 0.23	0.46 ± 0.24	0.46 ± 0.23	0.46 ± 0.22	0.46 ± 0.21	0.45 ± 0.25	0.45 ± 0.25	0.47 ± 0.23	0.46 ± 0.23	NS
Density [per min, μ ± σ ]	1.29 ± 2.44	1.87 ± 1.56	0.91 ± 1.36	1.60 ± 1.27	1.16 ± 1.95	2.01 ± 1.82	0.30 ± 0.51	0.64 ± 0.84	2.91 ± 2.52	4.21 ± 2.14	1.15 ± 2.06	1.86 ± 1.57	<0.05^a^^,^^b^^,^^c^^,^^GC^

Wilcoxon rank sum tests were used to test for significance between patients with Parkinson's disease (PD) and control subjects (C).

a significant for expert 1,

b significant for expert 2,

c significant for expert 3,

d significant for expert 4,

e significant for expert 5,

GC *significant for group consensus*.

The patients had significantly fewer spindles than the controls (*p*-value < 0.05). Ten patients and only four controls had less than 10 SS in the 40 epochs of N2 sleep that were assessed; four patients and 0 controls had no SS. Only 3 patients compared to 10 controls had more than 20 SS in the group consensus.

As a supplementary check, the significance tests were performed on SS identifications from each of the five experts individually. The maximum peak-to-peak amplitude was, for all five experts, both before and after removal of frequencies below 4 Hz, significantly different in patients with PD compared to controls. The duration and oscillation frequency were also significantly different between the two groups for 4/5 of the experts, and density significantly different between the two groups for 3/5 of the experts. The mean and standard deviations of the SS morphology measures and the results from the significance tests are summarized in Table 2.

**Figure 2:**
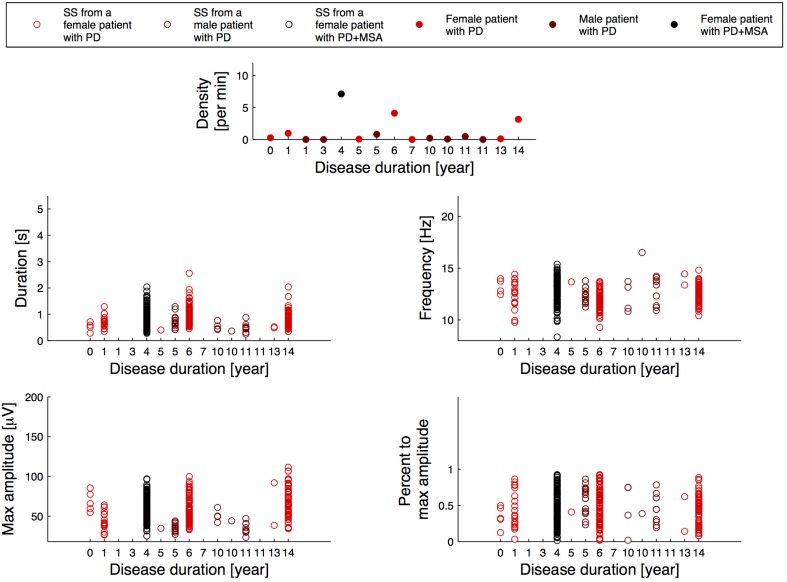
**Distribution of the morphology measures for the spindles from patients with Parkinson's disease (PD), where the patients are sorted according to their disease duration**. PD+MSA indicates a patient with PD, that later developed Multiple System Atrophy (MSA).

**Figure 3:**
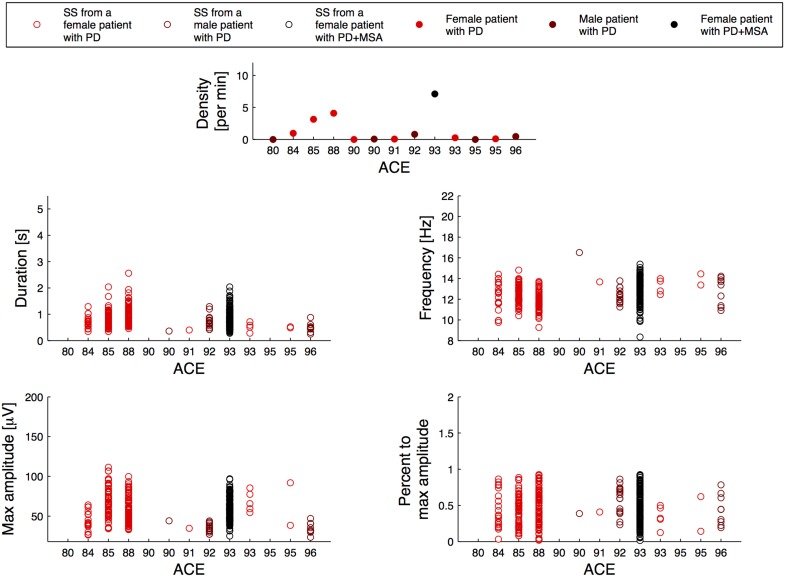
**Distribution of the morphology measures for the spindles from 13/15 patients with Parkinson's disease (PD), where the patients are sorted according to their Addenbrookse's cognitive examination (ACE) scores**. PD + MSA indicates a patient with PD, that later developed Multiple System Atrophy (MSA).

Figure 2 illustrates the relation between the SS measures and disease duration for the patients, and Figure 3 illustrates the relation between the SS measures and Addenbrooke's Cognitive Examination (ACE) score for the patients. Note that the x-axes are not continuous, but denote disease duration in years (Figure 2) and ACE score (Figure 3) for 15/15 and 13/15 of the patients, respectively. The three subjects with highest SS density are all females, and the one with the highest SS density is a patient with PD later diagnosed with MSA-P (indicated as PD+MSA in the figures). She is illustrated with black in Figures 1, 2, 3. No clear visual tendency between SS characteristics and disease duration or ACE score was seen for any of the measures. Supplementary Figure 1 illustrates the relation between SS measures and Hoehn and Yahr (H and Y) stage and Supplementary Figure 2 illustrates the relation between SS measures and the Unified Parkinson's Disease Rating Scale (UPDRS) Part III. No clear visual trends were seen.

Considering that the outlier PD patient with a very high spindle density (highest of all subjects in the study) later developed MSA, we reanalyzed the SS included in the group consensus when results from this outlier patient were left out, and found the same measures to be as significant different between the groups. Specifically, patients now have an even bigger decrease in SS density (−61.29%/−1.14 SS/min), a longer SS duration (+11.69%/+0.09 s), a slower frequency (−4.14%/−0.53 Hz) and a higher max peak-to-peak amplitude, both before (+16.93%/8.16 μV) and after (+17.95%/8.13 μV) removal of low frequencies when compared to controls. The results for this analysis are summarized in Table 3.

**Table 3:** **Mean (μ) and standard deviation (σ) for characteristics of spindles in patients with Parkinson's disease (PD) compared to controls (C)**.

**Spindle characteristic**	**Group consensus (759 SS)**		***P***
	**PD(-MSA)**	***C***	
Duration [sec, μ ± σ ]	0.86 ± 0.35	0.77 ± 0.36	<0.001
Frequency [Hz, μ ± σ ]	12.27 ± 1.07	12.80 ± 1.23	<0.001
Max peak-to-peak amplitude [μV, μ ± σ ]	56.35 ± 18.97	48.19 ± 15.55	<0.001
Max peak-to-peak amplitude After removal of frequencies < 4 Hz [μV, μ ± σ ]	53.42 ± 17.84	45.29 ± 14.41	<0.001
Percent-to-peak amplitude [μ ± σ ]	0.47 ± 0.23	0.46 ± 0.23	NS
Density [per min, μ ± σ ]	0.72 ± 1.28	1.86 ± 1.57	<0.007

*In this case, the patient that later was diagnosed with Multiple System Atrophy (MSA) was excluded from the PD group [PD (-MSA)]. P-values for the Wilcoxon rank sum tests between the two groups are shown. Only spindles in the group consensus are included in the comparison*.

**Figure 4:**
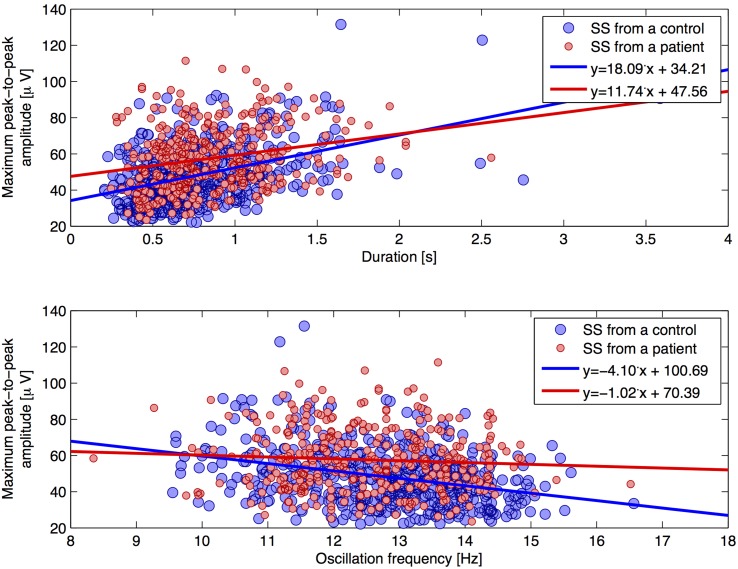
**Two scatterplots for individual SS characteristics**. The plot illustrates the maximum peak-to-peak amplitude (without removal of frequencies below 4 Hz) as a function of (1) duration (top plot) and (2) oscillation frequency (lower plot), respectively. Trend lines are added for each group.

Figure 4 shows scatterplots for the individual SS, where the maximum peak-to-peak amplitude (before removal of low frequencies) defines the y-axis and the oscillation frequency and duration defines the x-axis, respectfully. Linear trend lines are added on top of the scatterplots in order to see differences between groups. We found a trend of a positive correlation between the duration and maximum peak-to-peak amplitude. Interestingly, SS from patients showed this tendency to a lesser degree (slope of +11.74 μV/s) compared to SS from controls (slope of +18.09 μV/s). Also, we found a negative correlation of oscillation frequency and maximum peak-to-peak amplitude, and found this tendency to be less apparent for SS from patients (slope of −1.02 μV/Hz) compared to SS from controls (slope of −4.10 μV/Hz).

Table 4 summarizes the fraction of SS included in the group consensus that do not strictly pass AASM criteria for a spindle (11–16 Hz, 0.5–3.0 s). Overall, 25.3% of the SS identified by experts and included in the group consensus did not meet AASM criteria. Most of these “abnormal” SS would have been excluded because their duration is too short (16.9%) or have an oscillation frequency that is too slow (9.7%).

**Table 4:** **Percent of sleep spindles (SS) identified in the group consensus that do not strictly meet AASM criteria Iber et al. (2007)**.

**AASM criteria**	**Total SS**	**PD SS**	**PD-MSA SS**	**Control SS**	***P*-value PD vs. controls**	***P*-value PD-MSA vs. controls**
Duration too short (<0.5 s)	0.169	0.128	0.134	0.194	0.010	NS
Duration too long (>3 s)	0.001	0	0	0.002	NS	NS
Oscillation frequency too slow (<11 Hz)	0.097	0.090	0.099	0.101	NS	NS
Oscillation frequency too high (>16 Hz)	0.002	0.003	0.005	0.002	NS	NS
At least one criteria not met	0.253	0.212	0.228	0.278	0.027	NS

*There were a total of 344 SS from 11 patients with Parkinson's disease (PD) and 557 SS from 15 control subjects. There were 202 SS from 10 patients when one patient with PD, who later was diagnosed with Multiple System Atrophy (MSA) [PD(-MSA)] was left out. X^2^-tests were used to test for significance between spindles from PD patients (including and excluding the one with MSA) and control subjects*.

In order to determine if there was a difference between PD and controls in the frequency of “abnormal” spindles not meeting AASM criteria, we compared the groups. All 15/15 control subjects had SS, whereas only 11/15 patients with PD had some SS. It was found that control subjects show significantly more “abnormal” spindles not meeting AASM criteria, i.e., more spindles with a too short duration compared to patients with PD (Table 4). No significant difference was however found between groups when the outlier patient with PD + MSA was left out of the analysis.

When computing the SS characteristic based on AASM criteria, the same SS characteristics were found to be significantly different between PD patients and controls (Table 5). Analysis of these SS showed that patients with PD have a decreased density (−32.84%/−0.44 SS/min), and their SS are longer (+9.41%/+0.08 s), have a lower frequency (−2.69%/−0.35 Hz) and higher max peak-to-peak amplitude before removal of low frequencies (+21.34%/+10.37 μV) and after (+22.51%/+10.30) compared to controls. These differences are similar to those found based on all SS in the group consensus.

**Table 5:** **Mean (μ) and standard deviation (σ) for the spindle characteristics found for the spindles in the group consensus meeting the AASM criteria**.

**Spindle characteristic**	**Group consensus (673 SS)**		***P***
	***PD***	***C***	
Duration [sec, μ ± σ]	0.93 ± 0.33	0.85 ± 0.31	1.95 · 10^−4^
Frequency [Hz, μ ± σ]	12.65 ± 1.01	13.00 ± 0.96	9.04 · 10^−6^
Max peak-to-peak amplitude [μV, μ ± σ]	58.97 ± 16.64	48.60 ± 14.92	3.90 · 10^−16^
Max peak-to-peak amplitude After removal of frequencies < 4 Hz [μV, μ ± σ]	56.06 ± 15.75	45.76 ± 13.89	5.27 · 10^−18^
Percent-to-peak amplitude [μ ± σ]	0.47 ± 0.23	0.45 ± 0.23	NS
Density [per min, μ ± σ]	0.90 ± 1.71	1.34 ± 1.25	4.50 · 10^−2^

*Wilcoxon rank sum tests were used to test for significance between patients with PD and control subjects (C)*.

Table 6 summarizes inter-expert reliabilities of SS scoring, where the SS are grouped according to their confidence score. The mean inter-expert reliability of scoring “definite SS” computed by κ was found to be significant lower for patients compared to controls. Although not significant, a trend for a lower κ was found for “probable/definite SS” in patients compared to controls (*P* = 0.054). In all cases, the inter-expert reliability is lower for scoring SS in patients compared to controls.

**Table 6:** **Mean (μ) and standard deviation (σ) for the inter-expert reliability measure *F*_1_-scores and Cohen's Kappa (κ) for scoring sleep spindles (SS)**.

**SS group definition**	***F*_1_-score**		κ		***P***
	***PD***	***C***	***PD***	***C***	
Low confidence “maybe”	0.12 ± 0.11	0.17 ± 0.12	0.14 ± 0.11 “slight”	0.16 ± 0.12 “slight”	NS
Medium confidence “probably”	0.13 ± 0.10	0.19 ± 0.11	0.15 ± 0.10 “slight”	0.18 ± 0.11 “slight”	NS
High confidence “definitely”	0.24 ± 0.13	0.32 ± 0.13	0.21 ± 0.13 “fair”	0.32 ± 0.13 “fair”	4.76 · 10^−2^^κ^
Medium or high confidence “probably/definitely”	0.34 ± 0.15	0.39 ± 0.17	0.28 ± 0.15 “fair”	0.39 ± 0.17 “fair”	NS
All SS	0.41 ± 0.16	0.45 ± 0.15	0.32 ± 0.17 “fair”	0.43 ± 0.15 “moderate”	NS

The mean and standard deviations are taken across the ten expert-pairs available. Wilcoxon rank sum tests were used to test for significantly lower inter-expert reliability for scoring SS in patients with Parkinson's disease (PD) compared to control subjects (C).

κ *indicates significance for κ and F indicates significance for F_1_-score*.

## Discussion

Based on a group consensus of manually scored SS from five independent sleep experts, this study investigates morphological changes of SS in a central EEG lead of patients with PD compared to age- and sex-matched control subjects. The main findings of this study are that patients with PD have a decreased SS density, and that their SS have a longer duration, a slower oscillation frequency and higher maximum peak-to-peak amplitude. These results suggest that not only SS density but also specific morphological changes in SS have potential clinical utility when diagnosing PD. Further, the data suggests that the disease process affect directly or indirectly the brain regions responsible for the generation of SS. Future studies including more subtypes of PD and NDDs in general are however needed to investigate whether the specific morphological changes in SS can be used to differentiate different PD subtypes as well as different NDDs.

The results illustrate the fact that there are fewer SS in patients with PD, and that the few that are remaining are more pronounced when compared to those seen in controls. There could be several explanations for this. First, patients with PD have a more “blurred” EEG in general with either a lack of or an abnormal mixture of micro- and macro-sleep structures (Petit et al., 2004; Christensen et al., 2014b). This pattern may make it more difficult to identify distinct SS, as they would be buried within other undefined EEG microstructural changes. In this case, only the obvious SS would rise over background and be marked. Second, it could be that the neurodegenerative process has affected the thalamic neurons responsible for generating and controlling SS in such a way, that SS are only generated when very strong signals from pre-thalamic fibers reaches the thalamus resulting in more pronounced SS. Third, we cannot rule out that these SS changes could be the result of treatment with dopaminergic agents affecting the morphology of SS, although a previous report suggests that these drugs should increase spindle density (Puca et al., 1973), which is not what we observed.

It was found that patients with PD have a lower SS density compared to age and sex-matched controls. This finding is consistent with our and other groups' prior findings (Emser et al., 1988; Christensen et al., 2014a; Latreille et al., 2015), but contradicts those of other studies (Happe et al., 2004). According to Braak et al. (2003), the neurodegenarative progress in PD shows a progressive ascending course starting from the brain stem and spreading to additional brain structures. At some point, the neurodegeneration may affect or destroy the SS generator of the thalamus, resulting in fewer or no spindles. Interestingly, (Roth et al., 2000) found that medial thalamotomy abolishes spindle activity in N2 sleep systematically, but that pallido-thalamic tractotomy attenuate spindle activity only to a varying degree, with spindles reemerging after 3 months. It is therefore likely that neurodegenerative involvement of prethalamic fibers from the brain stem may affect spindle activity to a certain degree. In Figure 1, it is apparent that for four of the patients, no SS are included in the group consensus, and that for six other patients, less than 10 spindles were identified.

Surprisingly, a PD patient showing an abnormally high SS density was later diagnosed with MSA-P. Although only a single case, it is an interesting finding which support the hypothesis that spindles can be used as a marker of diagnostic subgroups of PD. Latreille et al. (2015) reported a decline in SS activity paralleling cognitive decline in patients with PD, suggesting that SS activity could be used as an early marker of Dementia. The number of patients included in present study is, however, too small to perform further subgroup analysis. Additionally, in both groups, younger subjects and females trend in showing slightly higher spindle densities when compared to older and male subjects. The three oldest male control subjects have negligible SS densities. These observations suggest that reduced SS density is not specific for PD, in agreement with the fact that many conditions such as cognitive function, memory consolidation, pharmacological interventions and pre-PSG conditions have been reported to influence SS density (De Gennaro and Ferrara, 2003; Caporro et al., 2012). Further analysis including more PD and iRBD patients, together with a more in-depth investigation of cognitive decline and disease severity would be needed to evaluate the relation of abnormalities in SS development in the disease process, and the use of SS as a prognostic marker. Additionally, SS density has also been reported decreased for other conditions such as Dementia, Alzheimer's disease (AD) and mild cognitive impairment (Rauchs et al., 2008; Westerberg et al., 2012; Latreille et al., 2015), and is also a sign of normal aging (Wauquier, 1993; De Gennaro and Ferrara, 2003; Ktonas et al., 2009).

To our knowledge, no studies have investigated the impact of L-DOPA on SS morphology. Previous studies have reported that SS density is increased in patients with PD taking dopaminergic treatment compared to non-treated patients, but the study lacks a comparison to controls, and evaluation of spindle morphology (Puca et al., 1973). As dopaminergic treatments were not discontinued in this study, we cannot rule out that the changes in SS morphology observed are due to the dopaminergic interactions from the treatments, although we do not believe so, as we did not see increases in SS density in these subjects. Future studies will have to investigate this further including a potential association between amount and duration of L-DOPA and/or dopamine agonist treatment and SS morphological changes.

Surprisingly, SS in patients with PD had a longer duration and a higher maximum peak-to-peak amplitude. To our knowledge, no other studies have reported differences in SS duration in patients with PD when compared to controls. The maximum peak-to-peak amplitude significantly differ for SS identifications in the group consensus as well as for each of the individual expert's identifications. This finding was also significant after we filtered the data to eliminate the impact of low frequency, high amplitude waves. This was surprising, and contradicts the idea that polygraphic features such as SS and K-complexes are less well formed in various NDDs (Petit et al., 2004; Ktonas et al., 2009). By computing maximum peak-to-peak amplitude both without any further filtration and after elimination of low frequencies, our data show that patients with PD show SS with higher amplitudes, regardless of the EEG patterns surrounding them. Margis et al. (2015) reports increased sigma power in N2 sleep of patients with PD vs. controls. Increased sigma power is consistent with our findings of increased duration and amplitude of spindles, which would overpower the decrease in spindle density we and others have reported in PD. Interestingly, SS morphology was unchanged in schizophrenia patients compared to controls, even though they had a significant decrease in SS density (Wamsley et al., 2012).

Enhanced maximum peak-to-peak amplitude is also not consistent with the findings of Latreille et al. (2015), who reports no significant differences of SS amplitude between PD patients and controls, and significantly reduced SS amplitude in patients with PD, who later developed Dementia when compared with controls. The SS in Latreille et al. (2015) were found automatically and mandated a duration criteria of least 0.5 s to be included. Also, the spindle detection method includes a filtration of the signal (11–15 Hz) and a threshold determined based on root-mean-square (RMS) values of the background NREM activity (Martin et al., 2013). Lastly, the SS in Latreille et al. (2015) were detected in all NREM stages, and the individual SS characteristics (amplitude and frequency) were computed as the mean of both hemispheres, as they found no significant hemispheric interaction. The definition of SS is thus not the same in the two studies, and the different results could be due to the fact that automatic detectors detect SS that humans cannot see. Another explanation could be that the detector in Latreille et al. (2015) lack to identify the smaller SS in controls, thereby enlarging the mean spindle amplitude in controls. If the threshold used is based on values across all NREM sleep stages, different amount of NREM stages between controls and patients influences the threshold, maybe resulting in harder thresholds to cross for control spindles. Lastly, taking into account the fact that PD patients show more mixed sleep patterns making sleep stages more difficult to distinguish (Danker-Hopfe et al., 2004; Jensen et al., 2010), it could also be that more N3 sleep is present in the annotated data of patients compared to controls, although we did select data from N2 sleep according to each hypnogram. Whether the contradicting findings are due to methodological reasons only, have to be investigated in future studies, e.g., by applying different automatic spindle detectors on the same dataset and on data from different derivations, and see if the morphological alterations are consistent across detectors, manually scorings and derivations.

EEG slowing has been frequently reported in PD (Petit et al., 2004; Rodrigues Brazète et al., 2013), including slowing in occipital, temporo-occipital and frontal regions (Sirakov and Mezan, 1963; Soikkeli et al., 1991; Primavera and Novello, 1992). It is therefore not surprising that we found slower SS oscillation frequencies in PD patients. Whether or not this is specific for PD or generalizable to other NDDs will need further investigations. In AD, Rauchs et al. (2008) found no change in spindle density but found that fast spindles (defined as having frequencies of 13–15 Hz) were significantly reduced when compared to age-matched controls. Consistently, Westerberg et al. (2012) found that patients with amnestic mild cognitive impairment had fewer N2 spindles compared to age-matched controls, and that the reduction was seen in fast spindles (13–15 Hz) and not in slow spindles (11–13 Hz). Latreille et al. (2015) found significant lower SS frequency in patients with PD who later developed Dementia compared to controls, but not in Dementia-free patients with PD compared to controls. This last study might however suffer from a selection bias as they automatically defined SS within a certain frequency range, as stated by the AASM. Nonetheless, as in this study, we found that PD patients had a slower SS frequency, both when looking at SS included in the group consensus, but also when looking at SS strictly meeting AASM criteria.

Figures 2, 3 and Supplementary Figures 1, 2 report on SS measures for the PD group consensus, but with subjects sorted according to their disease duration (Figure 2), their ACE score (Figure 3), their H and Y stage (Supplementary Figure 1) and UPDRS part III score (Supplementary Figure 2). Although no clear tendency was seen for any of the SS measures for disease duration, ACE score, H and Y stage or UPDRS part III score, longitudinal studies are likely needed to determine whether SS morphology measures can provide prognostic value. Indeed, the patients included here may have had a PD diagnosis for various amounts of time, and inter-subject variation of disease progression and severity makes such a relationship very complicated to analyze. ACE is a brief assessment of cognitive functions and is in this study used as a screening tool to determine Dementia, which none of the patients had at inclusion. A more in-depth examination of cognitive functions as well as a follow-up study of the patients is needed to determine the subject-specific progression and severity rate. These rates can be compared to the SS morphology measures to investigate the prognostic value.

A biomarker does not have to be specific to a disease to have clinical utility, and combining the different SS measures may reveal that different diseases show different trends or different combinations of changes in SS morphology measures. If a trend is found, it is important to also look at SS that might fall out of the stated AASM criteria, as not doing that may misrepresent the data. Table 4 shows that a rather high proportion of SS in both groups do not meet AASM criteria. Additionally, when looking at inter-expert reliability, it was found that experts are less likely to agree on definite SS in patients when compared to controls. Considering that automatic SS detectors are likely to be used in patients with NDDs, it is highly encouraged to build detectors capable of detecting atypical SS as well. Such atypical SS could be spindles with abnormal duration or frequency or spindles surrounded by EEG that is not typically seen in N2 sleep. Because of this, detectors should not be constrained or designed to perform well only in the context of a single expert or for normal EEG. Ideally, automatic detectors should give a confidence score for each detected SS and group subtypes of SS using specific parameters describing their morphology. Specifically, description of “probable SS” in different patient groups may give a better idea of the specific morphological changes that can be observed for each disease. Also, such studies should investigate how disease duration and/or severity impact morphology. Such in-depth studies would be beneficial to better understand the pathological differences between the NDDs and also see if any of the morphology measures hold potential for separating diseases or subtypes of them.

In conclusion, we investigated SS in an objective way and found that the oscillation frequency and duration of SS manually scored in clinical settings are not necessarily bound to the limits given by AASM. The shorter or slower SS must have had an ability to stand out from the background EEG, and we believe that these per-definition-not-SS should be included in studies analyzing SS morphology changes, particularly when searching for disease biomarkers.

Based on a group consensus of five individual experts' identification of SS in N2 sleep, we compared 15 patients with PD with 15 age-matched control subjects and found that patients show a lower SS density and that their SS have a longer duration, a higher maximum peak-to-peak amplitude and a slower oscillation frequency. All the included patients were taking dopaminergic treatment, and we can therefore not rule out that the significant differences found could be due to treatment effects. We conclude that SS are significantly altered in patients with PD, but that due to high inter-subject variability in disease progression and severity, future longitudinal studies are needed to investigate the clinical utility of the SS morphology changes as well as their value as prognostic biomarkers.

## Financial support

The PhD project is supported by grants from H. Lundbeck A/S, the Lundbeck Foundation, the Technical University of Denmark and the Center for Healthy Aging, University of Copenhagen.

### Conflict of interest statement

The Reviewer Veronique Latreille declares that, despite being affiliated to the same institution as the author Simon C. Warby, the review process was handled objectively and no conflict of interest exists. The authors declare that the research was conducted in the absence of any commercial or financial relationships that could be construed as a potential conflict of interest.

## Supplementary material

Supplementary Figure 1 **Distribution of the morphology measures for the spindles from 11/15 patients with Parkinson's disease (PD), where the patients are sorted according to their Hoehn and Yahr (H and Y) stage**.

Supplementary Figure 2 **Distribution of the morphology measures for the spindles from 11/15 patients with Parkinson's disease (PD), where the patients are sorted according to their Unified Parkinson's Disease Rating Scale (UPDRS) part III score**.

## Abbreviations

AASM: American Academy of Sleep Medicine
EEG: electroencephalography
iRBD: idiopathic REM sleep behavior disorder
MSA: Multiple System Atrophy
NDD: Neurodegenerative disorders
PD: Parkinson's disease
PSG: polysomnographic
REM: Rapid eye movements
SS: Sleep spindles.
