# Supplementary figures and images for: Sleep spindle alterations in patients with Parkinson's disease
Source: Front Hum Neurosci. 2015 May 1;9:233. doi: 10.3389/fnhum.2015.00233 (PMC4416460; doi:10.3389/fnhum.2015.00233)

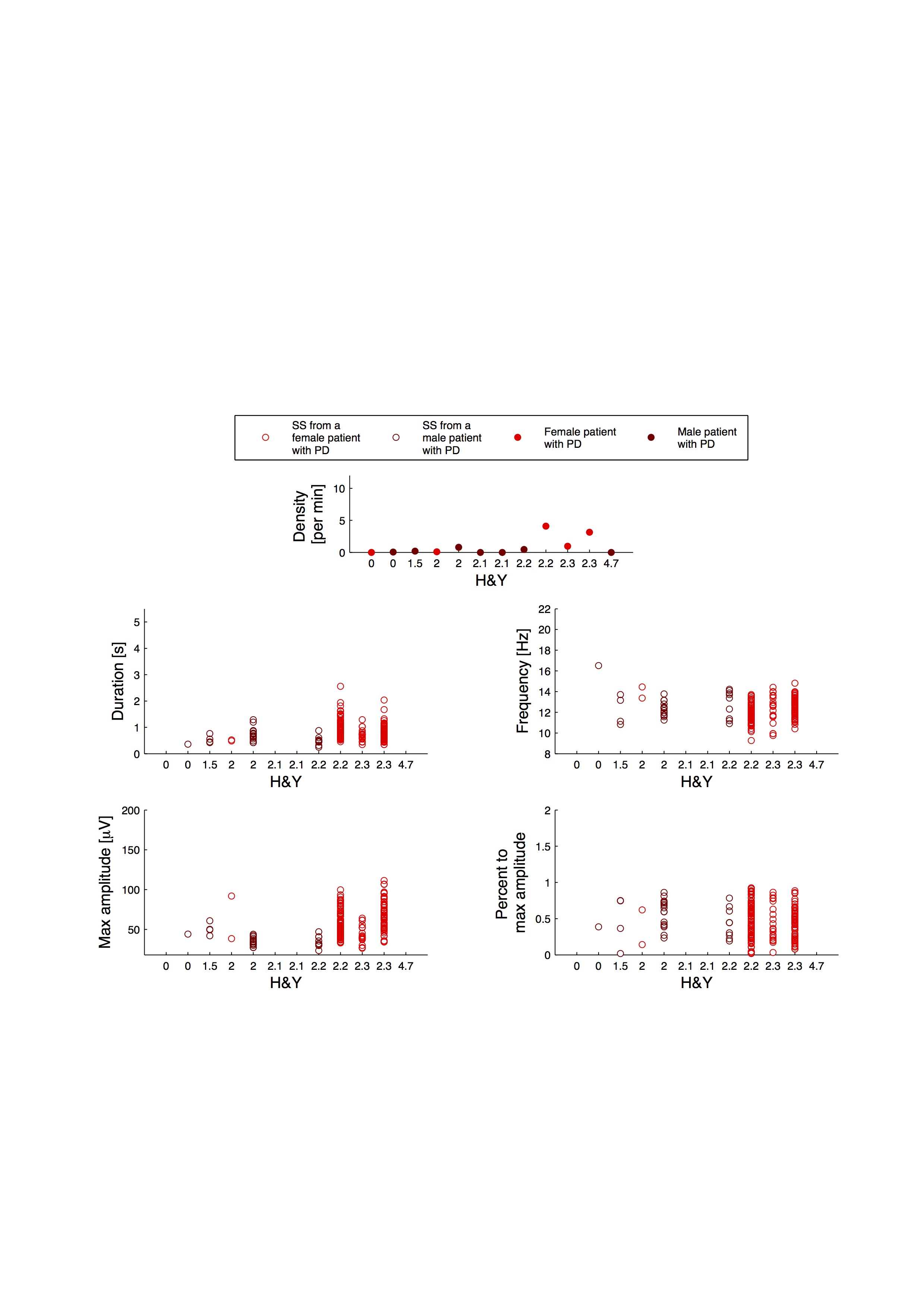

Supplement: Supplementary Figure 1 — Distribution of the morphology measures for the spindles from 11/15 patients with Parkinson's disease (PD), where the patients are sorted according to their Hoehn and Yahr (H and Y) stage. [file Image1.JPEG]

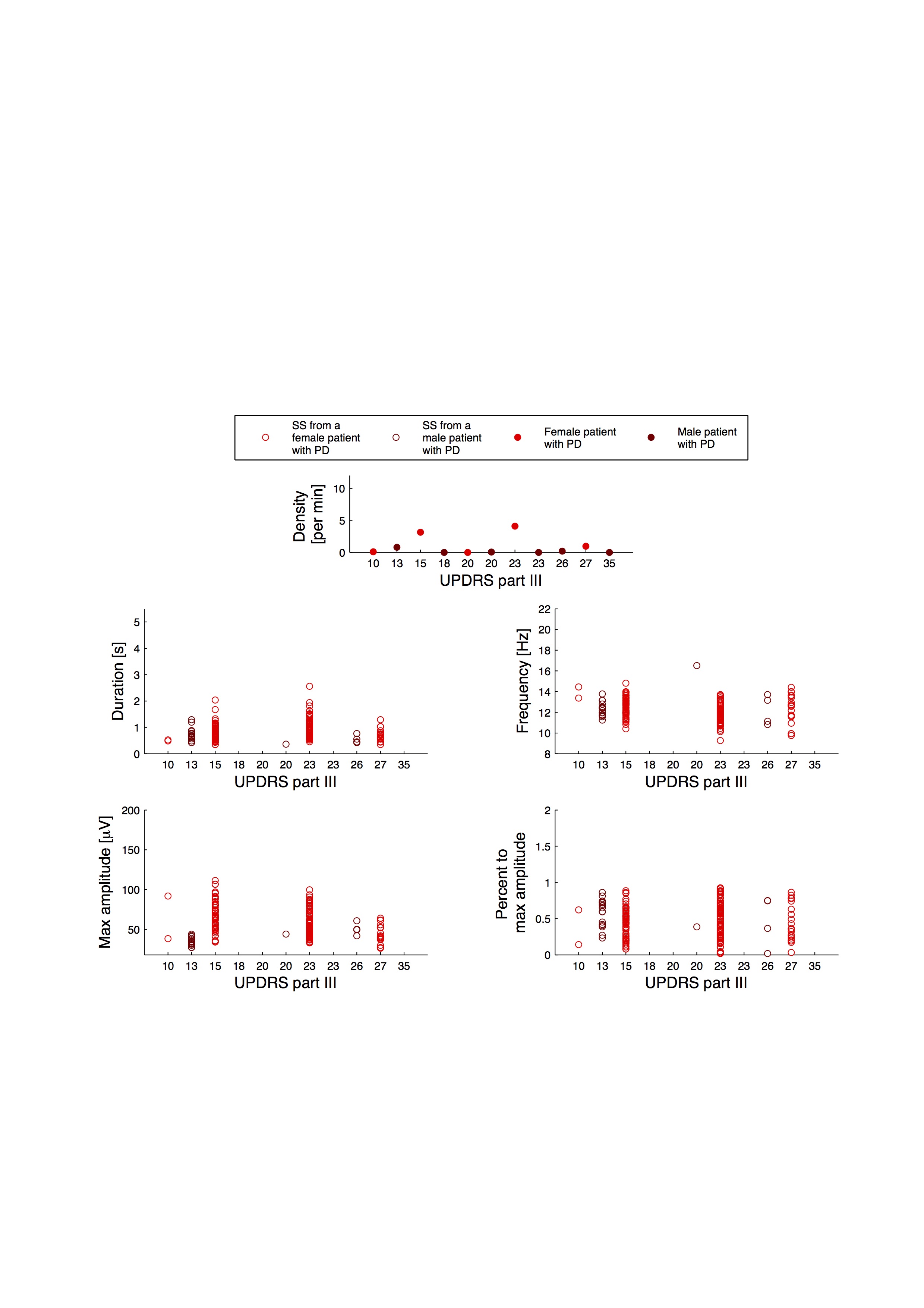

Supplement: Supplementary Figure 2 — Distribution of the morphology measures for the spindles from 11/15 patients with Parkinson's disease (PD), where the patients are sorted according to their Unified Parkinson's Disease Rating Scale (UPDRS) part III score. [file Image2.JPEG]
